# Relationship between Hospitalization-Associated Disability and Functional Recovery

**DOI:** 10.1016/j.arrct.2025.100529

**Published:** 2025-09-27

**Authors:** Haruka Adachi, Wataru Kozuki, Aki Gen, Ryo Tsujinaka, Tetsuya Ueda, Yumi Higuchi

**Affiliations:** a Graduate School of Rehabilitation Science, Osaka Metropolitan University, Osaka City, Osaka; b Department of Rehabilitation, Ikeda City Hospital, Osaka, Japan

**Keywords:** Early ambulation, Hospitalization-associated disability, Rehabilitation

## Abstract

**Objective:**

To investigate the relationship between the timing of functional recovery milestones and hospitalization-associated disability (HAD) in geriatric patients ≥70 years old.

**Design:**

Retrospective cohort study.

**Setting:**

A general acute care hospital.

**Participants:**

A total of 195 patients (N=195) aged ≥70 years (mean age 81.7±7.4y; 48.2% women) who were hospitalized for acute medical conditions. Patients with baseline disability or missing key data were excluded.

**Interventions:**

Not applicable.

**Main Outcome Measures:**

The HAD was defined as a ≥5-point decline in the Barthel Index compared to baseline functioning 2 weeks before admission. The relationship between HAD and the number of days to initiation of sitting, wheelchair transfer, and ambulation was examined.

**Results:**

The HAD occurred in 34.4% of patients. Those who developed HAD experienced significant delays in the initiation of sitting, wheelchair transfers, and ambulation. Multivariable logistic regression analysis showed that delayed initiation of wheelchair transfer and ambulation were independently associated with HAD, after adjusting for age, Barthel Index at admission, dementia, emergency admission, and total physical therapy time. Notably, the timing of ambulation initiation demonstrated moderate predictive ability for HAD (area under the curve=0.741; 95% CI, 0.656-0.825), with receiver operating characteristic analysis indicating a cut-off value of 1.5 days.

**Conclusions:**

Delayed initiation of wheelchair transfer and ambulation was associated with increased risk of HAD in older hospitalized patients. Early initiation of ambulation may serve as a key target for HAD prevention strategies.

## Highlights

• Hospitalization-associated Disability (HAD) occurred in 34.4% of hospitalized elderly patients.
• Delayed ambulation and wheelchair transfer were linked to HAD.
• Ambulation within 1.5 days may help prevent HAD.
• Early mobility interventions may improve outcomes and reduce costs.

Advancements in acute care medicine have significantly improved survival rates among patients with various acute illnesses. However, factors such as prolonged bed rest, limited physical activity, and the stress of adapting to an unfamiliar environment often contribute to hospitalization-associated disability (HAD). The HAD is defined as a decline in activities of daily living (ADL) by at least 1 level at discharge compared to prehospitalization status, despite resolution of the acute condition. Studies report that approximately 30% of hospitalized patients aged ≥70 years’ experience HAD.^1^^,^^2^ With the aging population and the rising proportion of older inpatients, there is growing concern about an increased incidence of HAD, which negatively impacts quality of life and long-term prognosis.^3^ This trend also carries the potential to raise overall health care and caregiving costs, making HAD a significant societal issue.

Once HAD develops, recovery is often difficult,^4^ as it is associated with increased caregiving burden, poor prognosis, higher rates of hospital readmission, and mortality.^1^ Therefore, preventing HAD during hospitalization should be considered a key primary prevention strategy to improve post-acute outcomes. Since its initial definition in 2011, several studies have investigated risk factors for HAD. According to a meta-analysis by Hao et al,^5^ advanced age, female sex, number of comorbidities, prehospital ADL status, dementia, and prolonged hospital stay have been identified as major risk factors. Additionally, polypharmacy, nutritional status, physical inactivity, delirium,^5^ surgical interventions,^6^ catheter placement, and insufficient involvement of physical therapists^7^ contribute to ADL decline during hospitalization.

In acute care hospitals, where patients are treated for acute illnesses, traumatic injuries, or postoperative conditions, early intervention by physiotherapists is crucial to prevent physical deconditioning. This includes reducing physical inactivity, promoting early mobilization, and increasing activity levels. Rehabilitation programs must be implemented with careful risk management after physical therapy guidelines and disease-specific recommendations. Previous studies^8^ have demonstrated that early mobilization—initiated after confirming basic hemodynamic stability and safety—can accelerate functional recovery, even in critically ill patients.

In this study, the process of functional recovery was defined as "the progression of increasing activity levels through early mobilization from bed," specifically focusing on milestones such as the initiation of sitting, transfer to a wheelchair, and ambulation. The dates of these milestones were retrospectively extracted from standardized physiotherapy notes, which were completed after each inpatient session conducted 5 d/wk. Although the importance of early rehabilitation for preventing HAD has been emphasized, its specific relationship with functional recovery remains unclear. This study aimed to investigate the relationship between HAD and functional recovery.

## Methods

### Study design

This retrospective, single-center observational study was conducted at a general acute care hospital in Japan. We reviewed patient medical records and adhered to the principles of the Declaration of Helsinki and the ethical guidelines for medical research established by the Ministry of Health, Labor, and Welfare. The study protocol was approved by the ethics committees (No. 3492) and the University (No. 2023-121). The participants were informed about the study through an opt-out consent process.

### Participants

From August 2022 to July 2023, we targeted patients aged 70 years and older who were hospitalized for acute illnesses. Based on a previous study,^1^ we excluded patients with conditions that directly cause motor dysfunction, such as fractures and stroke, those with poor prognoses, such as terminal cancer, patients with a preadmission Barthel index score of 0, patients transferred from other hospitals, and patients admitted to the intensive care unit to eliminate the influence of intensive care unit-acquired weakness.

### Definition of HAD

The Barthel Index^9^ assesses 10 categories of ADL, including eating, transferring, dressing, toileting, bathing, walking, climbing stairs, grooming, and bladder and bowel control, with scores ranging from 0 to 100. Higher scores indicate significant independence. The HAD was defined as a decrease of 5 or more points in the Barthel index score between a stable state 2 weeks before hospitalization and discharge, based on established criteria.^1^ Retrospective reports of ADL 2 weeks before hospitalization are reportedly highly reliable,^10^ and they were obtained by nurses or physical therapists in this study. When the patients could not provide information, data were collected from their key caregivers or care managers. ADL assessment at discharge was conducted by a physiotherapist.

### Collected information

The following data were extracted from the participants’ medical records.

#### Clinical characteristics

Clinical characteristics, including age, sex, body mass index, residence at home before admission, length of hospital stay, and discharge to home, were obtained.

#### Medical characteristics

The following medical characteristics were collected at admission: primary diagnosis; presence or absence of surgical treatment; presence of dementia or delirium; use of continuous drip infusion; urinary catheter, or drain; emergency admission status; Mini Nutritional Assessment–Short Form score; laboratory data at admission (serum albumin, hemoglobin, and C-reactive protein); number of comorbidities; and number of medications.

#### Functional characteristics

Data on functional characteristics included Barthel index scores (before admission, at discharge), number of days from admission to rehabilitation initiation, number of days from rehabilitation prescription to initiation, and total and daily physical therapy time.

#### Functional recovery process

In addition, as part of the functional recovery process, data were collected on the days when sitting, wheelchair transfer, and ambulation were initiated. These milestone dates were retrospectively extracted from standardized physiotherapy notes in the electronic medical record, which are completed after each therapy session routinely conducted 5 d/wk.

### Physical therapy for hospitalized patients

All participants underwent standard physical therapy based on disease-specific rehabilitation guidelines and the second edition of the Physical Therapy Guidelines issued by the Physical Therapy Association.^11^ Once hemodynamic stability was achieved, the therapy began with low-intensity exercises in bed, followed by bedside sitting, wheelchair transfer, and walking. Strength and endurance training using resistance bands, treadmills, or ergometers was also incorporated as appropriate.

### Statistical analysis

Clinical, medical, and rehabilitation-related characteristics were compared between the HAD and non-HAD groups. Continuous variables were summarized as means ± standard deviation and compared using either a 2-sample *t* test or the nonparametric Mann–Whitney *U* test, depending on data normality, which was assessed using the Shapiro–Wilk test. Categorical variables were analyzed using chi-square tests. Logistic regression analysis was performed to identify independent predictors of HAD, adjusting for confounding factors based on prior literature and clinical relevance. Multicollinearity among independent variables was assessed using the variance inflation factor; all variables had variance inflation factor values below 2, indicating no meaningful collinearity. Receiver operating characteristic curve analysis was conducted to assess the diagnostic ability of functional recovery factors, with cut-off values determined using the Youden Index. Statistical significance was set at *P* < .05, and the analyses were performed using SPSS version 29.^a^

## Results

### Clinical characteristics

**Fig 1:**
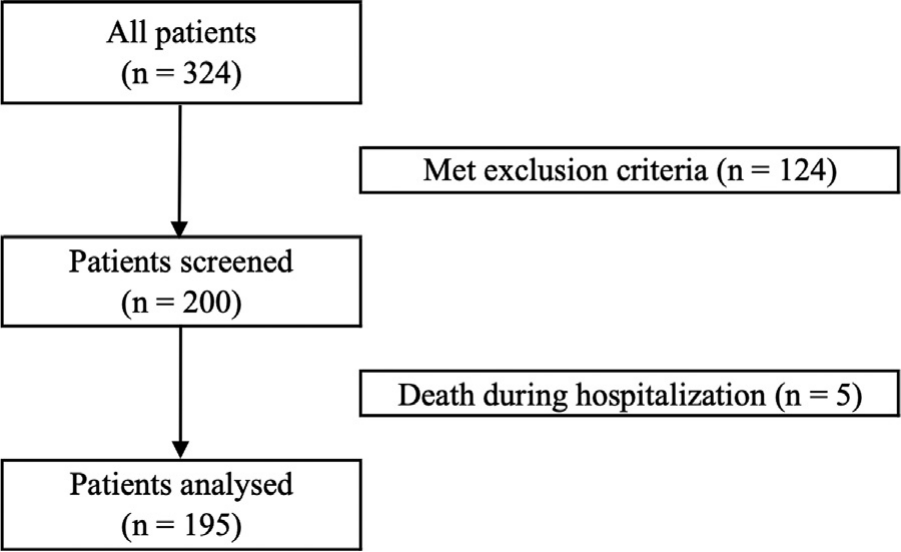
Flow diagram of study participants.

A total of 324 patients were hospitalized for acute illnesses and received rehabilitation between August 2022 and July 2023. Of these, 195 patients comprised the final analytical cohort. Exclusions resulted from meeting predefined criteria or death during hospitalization (see fig 1 for details. The mean age of the analyzed population was 81.7±7.4 years, with 48.2% women and a mean body mass index of 21.7±3.8 kg/m². The most common acute admission diagnoses included malignancies requiring surgery or chemotherapy; respiratory infections such as acute pneumonia; gastrointestinal emergencies including ileus or gastrointestinal bleeding; musculoskeletal disorders requiring surgical intervention (eg, joint replacement or spinal canal decompression); renal/urological conditions such as urinary tract infection; and cardiovascular events such as acute heart failure. The mean length of hospital stay was 18.6±14.3 days, and 93.3% of patients were discharged home.

The mean Barthel index score declined from 85.5±24.1 before admission to 79.3±27.5 at discharge. The total average duration of physical therapy was 3.7±3.8 hours, with a daily average of 0.4±0.1 hours. Regarding the functional recovery process, the mean initiation days from the start of rehabilitation were 1.4 ± 1.2 days for sitting, 1.7±1.6 days for wheelchair transfer, and 2.0 ± 1.8 days for ambulation.

### Patient characteristics based on the presence or absence of HAD

Among the 195 patients, 67 (34.4%) were diagnosed with HAD. The clinical characteristics of the patients are shown in table 1, their medical characteristics are shown in table 2, and their functional characteristics are shown in table 3. Patients with HAD were significantly older (*P*=.03), had longer hospital stays (*P*<.01), and were less likely to be discharged (*P*<.01) than those without HAD. Additionally, dementia was more prevalent in the HAD group (*P*<.01), and a higher proportion of these patients were admitted through the emergency department (*P*=.03). The Barthel index score at discharge was also significantly lower in the HAD group (*P*<.01).

**Table 1:** Patient clinical characteristics.

Characteristic	All	HAD	Non-HAD	*P* Value
	(N=195)	(n=67)	(n=128)	
**Age, y**	81.7±7.4	82.6±9.6	81.3±5.9	.03
**Female, n (%)**	94 (48.2)	38 (56.7)	56 (43.8)	.09
**BMI (kg/m^2^)**	21.7±3.8	21.4±4.4	21.8±3.5	.46
**Living at home, n (%)**	171 (87.7)	55 (82.1)	116 (90.6)	.09
**LOS, d**	18.6±14.3	23.9±20.1	15.8±8.9	<.01
**Home discharge, n (%)**	182 (93.3)	55 (82.1)	127 (99.2%)	<.01

Values are presented as means ± standard deviation or number (%). Differences between the HAD and non-HAD groups were analyzed using *t* test, Mann–Whitney *U* test, and chi-square test.

Abbreviations: BMI, body mass index; LOS, length of stay.

**Table 2:** Medical characteristics of patients.

Characteristic	All	HAD	non-HAD	*P* Value
	(N=195)	(n=67)	(n=128)	
**Main admission diagonosis**				
Cancer, n (%)	58 (29.7)	16 (23.9)	42 (32.8)	.20
Respiratory, n (%)	53 (27.2)	20 (29.9)	33 (25.8)	.54
Digestive, n (%)	21 (10.8)	6 (9.0)	15 (11.7)	.55
Musculoskeletal, n (%)	20 (10.3)	5 (7.5)	15 (11.7)	.35
Renal/urologic, n (%)	16 (8.2)	8 (11.9)	8 (6.3)	.17
Circulatory, n (%)	9 (4.6)	6 (9.0)	3 (2.3)	.07
Other, n (%)	18 (9.2)	6 (9.0)	12 (9.4)	.92
**Surgery, n (%)**	49 (25.1)	14 (21.0)	35 (27.3)	.18
**Dementia,n (%)**	55 (28.2)	28 (41.8)	27 (21.1)	<.01
**Delirium, n (%)**	36 (18.5)	17 (10.4)	19 (14.8)	.07
**Continuous drip infusion, n (%)**	133 (68.2)	47 (70.1)	86 (67.2)	.67
**Urinary catheter, n (%)**	53 (27.2)	20 (29.9)	33 (25.8)	.54
**Drain, n (%)**	37 (19.0)	11 (16.4)	26 (20.3)	.51
**Emergency admission, n (%)**	155 (79.5)	59 (88.1)	96 (75.0)	.03
**MNA-SF score at admission, points**	9.6±3.1	9.2±3.1	9.9±3.1	.10
**Laboratory data at admission**				
Serum albumin, g/dL	3.4±0.6	3.3±0.7	3.4±0.5	.29
Hemoglobin, g/dL	11.5±2.1	11.4±2.0	11.6±2.2	.52
C-reactive protein, mg/dL	5.0±5.7	5.8±6.4	4.5±5.2	.15
**Number of cormobidities, n**	1.3±1.0	1.3±1.0	1.3±1.1	.98
**Number of medications, n**	6.7±3.9	6.3±3.5	6.9±4.0	.40

Values are presented as means ± standard deviation or number (%). Differences between the HAD and non-HAD groups were analyzed using *t* test, Mann–Whitney *U* test, and chi-square test.

Abbreviation: MNA-SF, mini nutritional assessment - short form.

**Table 3:** Functional characteristics of patients.

Characteristic	All	HAD	non-HAD	*P* Value
	(N=195)	(n=67)	(n=128)	
**Barthel Index score**				
Before admission, points	85.5±24.1	81.4±26.2	87.6±22.7	.08
At discharge, points	79.3±27.5	63.1±29.0	87.7±22.6	<.01
**From admission to start of rehabilitation, d**	4.6±4.7	4.7±5.6	4.6±4.2	.79
**From prescription to start of rehabilitation, d**	1.3±0.8	1.3±0.7	1.3±0.8	.69
**Total physical therapy time, h**	3.7±3.8	4.9±5.2	3.0±2.6	.01
**Physical therapy time/d, h**	0.4±0.1	0.4±0.2	0.4±0.1	.35
**Functional recovery process**				
Initiation of sitting, d	1.4±1.2	1.6±1.6	1.2±0.9	.02
Initiation of wheelchair transfer, d	1.7±1.6	2.4±2.3	1.3±0.9	<.01
Initiation of ambulation, d	2.0±1.8	3.1±2.4	1.5±1.2	<.01

Values are presented as means ± standard deviation. Differences between the HAD and non-HAD groups were analyzed using Mann–Whitney *U* test.

Regarding the functional recovery process, the HAD group showed a significant delay compared to the non-HAD group in all measured aspects: initiation of sitting (1.6±1.6 vs 1.2±0.9d, *P*=.02), initiation of wheelchair transfer (2.4±2.3 vs 1.3±0.9d, *P*<.01), and initiation of ambulation (3.1±2.4 vs 1.5±1.2d, *P*<.01).

### Predictors of HAD occurrence during functional recovery

Logistic regression analysis identified delayed initiation of wheelchair transfer (odds ratio = 1.539, 95% CI, 1.172-2.020] and delayed initiation of ambulation (odds ratio = 1.786, 95% CI: 1.362-2.343) as independent predictors of HAD. Even after adjusting for age, Barthel index score at admission, dementia, emergency admission, and total physical therapy time, these factors remained significant (table 4).

**Table 4:** Logistic regression analysis of predictors of HAD occurrence on functional recovery process.

Model	Dependent Variable	B	OR	95% CI	*P* Value
Model 1	**Initiation of sitting**	0.226	1.253	0.934-1.681	.132
Model 2	**Initiation of wheelchair transfer**	0.431	1.539	1.172-2.020	.002
Model 3	**Initiation of ambulation**	0.580	1.786	1.362-2.343	<.001

Adjusted for age, Barthel Index, dementia, emergency admission, and total physical therapy time.

Abbreviation: OR, odds ratio.

**Fig 2:**
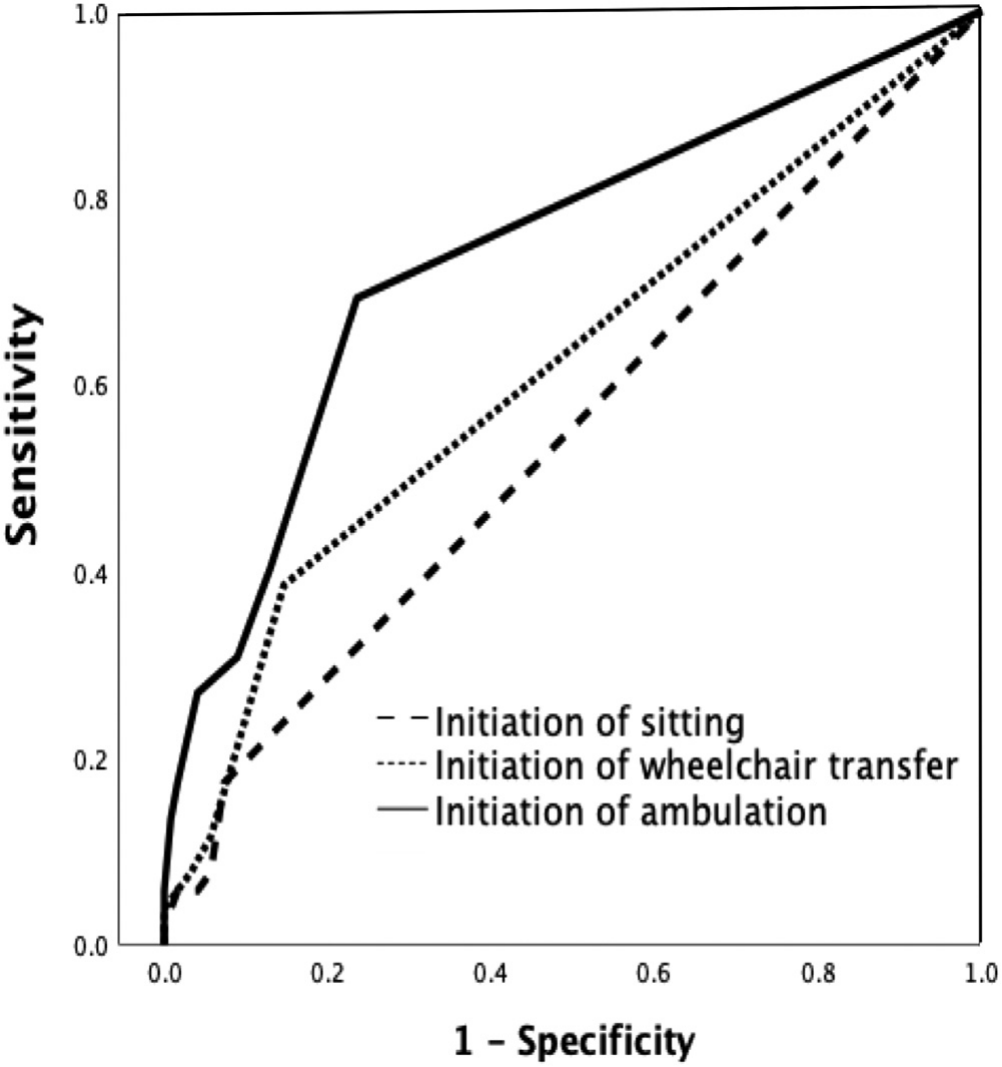
ROC curve of functional recovery process on HAD occurrence. Initiation of ambulation showed the highest predictive power (AUC=0.741; 95% CI, 0.656-0.825), followed by initiation of wheelchair transfer (AUC=0.617) and initiation of sitting (AUC=0.549). Abbreviations: ROC, receiver operating characteristic.

Receiver operating characteristic curve analysis demonstrated that the initiation of ambulation had the highest predictive power for HAD occurrence, with an area under the curve (AUC) of 0.741 (95% CI, 0.656-0.825). In contrast, the initiation of sitting (AUC=0.549; 95% CI, 0.453-0.645) and wheelchair transfer (AUC=0.617; 95% CI, 0.522-0.713) showed weaker predictive abilities (fig 2). The Youden Index identified an optimal cut-off of 1.5 days for ambulation initiation, yielding a sensitivity of 0.76 (95% CI, 0.64-0.86), specificity of 0.73 (95% CI, 0.65-0.81), positive predictive value of 0.60 (95% CI, 0.49-0.70), and negative predictive value of 0.85 (95% CI, 0.77-0.91), indicating moderate diagnostic accuracy.

## Discussion

The results of this study confirmed that wheelchair transfer and ambulation initiation, as components of the functional recovery process, remained significant independent factors associated with HAD, even after adjusting for age, Barthel index score at admission, dementia, emergency admission, and total physical therapy time. Additionally, ambulation initiation demonstrated moderate predictive ability for HAD, with a suggested cut-off of 1.5 days. These findings suggest that HAD is more likely to occur in patients who reach lower functional milestones during physical therapy.

To the best of our knowledge, this is the first study to investigate the association between HAD and functional recovery in patients hospitalized for acute illnesses. Among the 195 participants, 67 (approximately one-third) developed HAD. This prevalence rate is comparable to that reported in previous studies.^1^^,^^2^ Compared with patients without HAD, those with HAD were older, had a higher proportion of emergency admissions and dementia, exhibited lower Barthel index scores at discharge, experienced longer hospital stays, and had a lower rate of discharge to home. These characteristics align with the risk factors reported in existing meta-analyses,^2^^,^^3^^,^^5^ including advanced age, dementia, and prolonged hospital stay, supporting the findings of this study. Furthermore, HAD has been identified as a barrier to home discharge in previous research,^1^ consistent with the trends observed in the present study. Notably, patients with HAD exhibited a higher proportion of emergency admissions, which may reflect acute exacerbation or severity of illness. This finding aligns with those of prior studies^12^ reporting that emergent patients are more likely to experience physical function decline, suggesting a potential effect on HAD occurrence.

In this study, no significant differences were observed between the HAD and non-HAD groups in terms of time to rehabilitation initiation, total rehabilitation duration, or daily physical therapy time. At our institution, inpatient rehabilitation is initiated once the attending physician issues an electronic referral after evaluating the patient’s functional status and medical stability. However, significant delays in the initiation of sitting, wheelchair transfer, and ambulation were observed in the HAD group. Although previous studies have reported delays in sitting, standing, and ambulation initiation in patients with HAD after cardiac surgery,^13^ these factors were not analyzed using multivariate analysis. Although the current cohort consisted of older adults with a range of acute medical and surgical diagnoses—enhancing the external generalizability of the findings—this heterogeneity may also limit disease-specific applicability.

Logistic regression analysis identified delays in wheelchair transfer initiation and ambulation as independent factors associated with HAD. Early mobilization and initiation of ambulation have been reported to contribute to improvements in ADL in patients with stroke^14^ and those undergoing orthopedic surgery.^15^ In this study, the identification of initiation of ambulation as a predictive factor for HAD underscores its significance in HAD screening. Moreover, the establishment of 1.5 days as a specific cut-off for ambulation initiation facilitates the early identification of high risk patients, highlighting the clinical importance of early rehabilitation. This finding emphasizes the need for timely intervention within a limited hospitalization period in acute care hospitals.

### Study limitations

This study has some limitations. First, as a retrospective observational study, causal relationships cannot be established. Selection bias may have occurred as a result of the exclusion criteria, and information bias may have arisen from the retrospective review of medical records. These factors should be considered when interpreting the findings. Second, the participants were limited to patients admitted to a single institution, which restricts the generalizability of our findings. Third, the relatively small sample size may have limited the statistical power to detect smaller effects or differences between subgroups. Additionally, the 1-year observation period may not adequately capture longer-term temporal trends in the incidence of acute illness or their potential influence on HAD. Furthermore, potential influencing factors, such as disease-specific characteristics and psychological and social factors, were not assessed. Future studies must develop a predictive diagnostic model for HAD based on the functional recovery process and known risk factors. Furthermore, establishing intervention strategies based on the early identification of patients at high risk for HAD is crucial.

## Conclusions

This study highlights the importance of risk factors such as advanced age, emergency admission, and dementia in the development of HAD. Early initiation of ambulation may play a key role in preventing HAD during functional recovery. The established cut-off value of 1.5 days for ambulation initiation may serve as a useful criterion for the early identification of patients at high risk for developing HAD.

## Supplier

a. SPSS, version 29; IBM.
